# Supplementary material for: Helical-caging enables single-emitted large asymmetric full-color circularly polarized luminescence
Source: Nat Commun. 2024 Jan 4;15:251. doi: 10.1038/s41467-023-44643-8 (PMC10767107; doi:10.1038/s41467-023-44643-8)
Supplement: Supplementary file 1 — Supplementary Information [file 41467_2023_44643_MOESM1_ESM.pdf]

Supplementary Information

for

Helical-caging enables single-emitted large asymmetric full-color circularly polarized luminescence

Yajie Zhou[1], Yaxin Wang[1], Yonghui Song[2,3], Shanshan Zhao[1], Mingjiang Zhang[1], Guangen Li[1], Qi Guo[1], Zhi Tong[1], Zeyi Li[1], Shan Jin[4,5,6], Hong-Bin Yao[2,3], Manzhou Zhu[4,5,6], Taotao Zhuang[1,2*]

1. Department of Chemistry, University of Science and Technology of China, Hefei, 230026, PR China.

2. Hefei National Research Center for Physical Sciences at the Microscale, University of Science and Technology of China, Hefei, 230026, PR China.

3. Department of Applied Chemistry, University of Science and Technology of China, Hefei 230026, PR China.

4. Department of Chemistry and Centre for Atomic Engineering of Advanced Materials, Anhui University, Hefei, 230601, PR China.

5. Key Laboratory of Structure and Functional Regulation of Hybrid Materials of Ministry of Education, Anhui University, Hefei, 230601, PR China.

6. Key Laboratory of Functional Inorganic Material Chemistry of Anhui Province, Anhui University, Hefei, 230601, China.

*E-mail: tzhuang@ustc.edu.cn (T. Z.)

Table of Contents

Supplementary Methods……………………………………………………………………...…… 3
Supplementary Fig. 1……………………………………………………………………………….5
Supplementary Fig. 2……………………………………………………..………………………6
Supplementary Fig. 3……………………………………………………………….……………7
Supplementary Fig. 4……………………………..……………………………………………8
Supplementary Fig. 5…………………………………………………………………….………9
Supplementary Fig. 6……………………………………………..………………………10
Supplementary Fig. 7……………………………………………………...…………………11
Supplementary Fig. 8…………………………………………………………...……………12
Supplementary Fig. 9……………………………………………....……………………13
Supplementary Fig. 10………………………………………………………….……………14
Supplementary Fig. 11…………………………………………………………….……………15
Supplementary Fig. 12…………………………………………………………………….………16
Supplementary Fig. 13…………………………………………………………………….………17
Supplementary Fig. 14……………………………………………………………………….…18
Supplementary Fig. 15……………………………………………….…………………………19
Supplementary Fig. 16…………………………………………………………………….………20
Supplementary Fig. 17…………………………………………………………….……………21
Supplementary Fig. 18……………………………………………………………………….…22
Supplementary Fig. 19…………………………………………………………….……………23
Supplementary Fig. 20…………………………………………………………………….………24
Supplementary Fig. 21………………………………………………………….………………25
Supplementary Fig. 22……………………………………………………………………………..26
Supplementary Fig. 23……………………………………..……………………………………27
Supplementary Fig. 24……………………………………………………….…………………28
Supplementary Table 1……………………………………………………….…………………29
Supplementary Fig. 25…………………………………………………………………….………30
Supplementary Fig. 26……………………………………………………….……………….…31
Supplementary References…………….…………………….………………….…………………32

Supplementary Methods

Chemicals and reagents

All reagents were used as received without further experimental purification.

Oleic Acid (OA, 90%+), Oleylamine (OLA, 90%+), 1-Dodecanethiol (DDT, 98%) and Zinc Acetate ($Zn(Ac)_2$, 99.5%+) were purchased from Adamas-beta. 1-Octadecene (ODE, >90%), Zinc Stearate ($ZnSt_2$) and Cuprous Iodide (CuI, 99.95%) were purchased from Aladdin. Gallium(III) Iodide (GaI_3, 99.999%) was purchased from Alfa Aesar. 10,12-Tricosadiynoic Acid (TDA, 98%+) were purchased from Macklin. Pentaerythritol tetrakis(3-mercaptopropionate) (PETMP), 2,2'-(Ethylenedioxy)diethanethiol) (EDDET), dipropylamine (DPA) and Irgacure 651 (I-651) were purchased from Sigma-Aldrich. Liquid crystal 5CB, E7, RM82, RM257 and chiral dopants (S/R811, S/R5011 and S/R1011) were purchased from Shijiazhuang Yesheng Chemical Technology Co., Ltd.

Synthesis of the white liquid crystal polymer

The preparation procedure of the white liquid crystal polymer is described below[1]: 283 mg RM257 and 5.5 mg R/S5011 were mixed with 100 mg toluene and water-bath heated to 80 °C for 5 min and cooled to 25 °C. Then, 16.45 mg PETMP, 34.8 mg EDDET and 2 mg I-651 were added into the solution. Lastly, 105 mg DPA (diluted to 1:100 ratio with toluene) and 20 μL the as-prepared Cu-Ga-S/ZnS QD toluene solution were added and the solution was stirred for 5 min, vacuum degassing for 1 min and poured onto a glass substrate for 24 h at 25 °C. Finally, the white liquid crystal polymer was irradiated with 365 nm UV light for 5 min.

Preparation of liquid crystal cells for spiral full-color emission generator

Uniform glass beads (Diameter = 25.0 μm) were dispersed in the UV-curable adhesive by ultrasonic dispersion. The mixture was placed between two glass sheets (The area is 4 cm^2) along the edge, and then irradiated by a UV lamp for 30 s to make the two glass sheets stick firmly. After that, SFEG was filled into cells via capillarity[2].

Fabrication of electroluminescent CPL device

The cleaned patterned indium tin oxide (ITO) glass substrate was ozone-treated for 2 min. The

PEDOT:PSS hole injection layer was spin-casted at 3000 rpm for 60 s and then baked at 150 °C for 30 min in air. The hole transport layer was formed on top of the PEDOT:PSS layer by spin-casting (3000 rpm, 60 s) the solution of 0.05 g PVK dissolved in 5 mL of chlorobenzene and baked at 150 °C for 30 min. Then, the QD emitting layer was formed by spin-casting (2000 rpm, 20 s) an octane dispersion of the white quantum dots (WQDs) (4 mg/mL) and drying at room temperature. Subsequently, the MgZnO NP inorganic electron transport layer was spin-deposited (1500 rpm, 60 s) with an ethanol dispersion of 30 mg/mL and then baked at 60 °C for 18 min. Finally, 100 nm-thick Al top cathode was formed by a thermal evaporation[3].

Preparation of freestanding liquid crystal films

The two pieces of glasses were assembled in an antiparallel manner to yield planar alignment condition and the cell gap was controlled by adding spacers. All the films were prepared inside the as-prepared cells. The mixture containing 460.5 mg nematic monomer (RM82), 39.5 mg chiral dopant (R/S1011), and 2.5 mg I-651 was weighed in a 2 mL vial, heated to 120 °C and then entered the cell through capillary action. Finally, the cell was photopolymerized under 365 nm at 95 °C for 40 min and the liquid crystal film was obtained[4].

Preparation of TDA films

The uniformly cut glasses were cleaned with ethanol and acetone and dried overnight in a vacuum drying oven. TDA was dissolved with the mixture of ethanol and deionized water (ethanol: deionized water = 4:1), and then gently dropped onto glass sheets. The glass sheets with TDA liquid drops were placed in a constant temperature and humidity incubator to dry into films before polymerization[5].

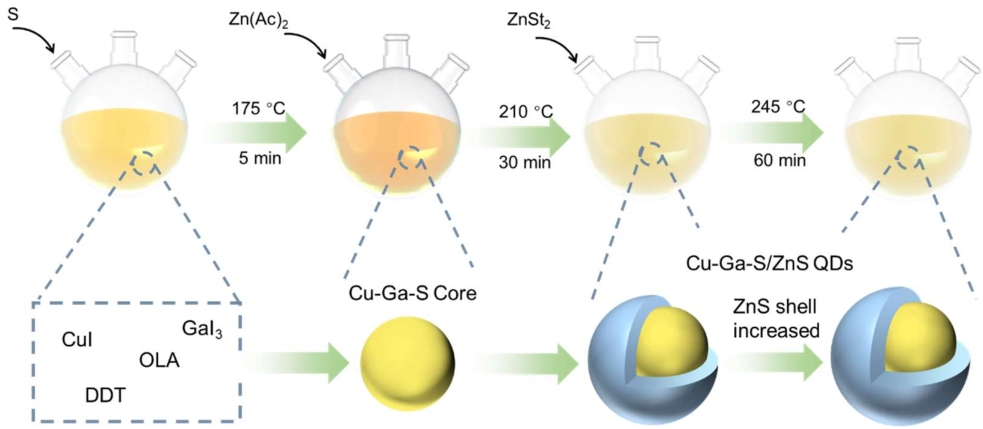

Supplementary Fig. 1 | Synthesis of the white quantum dots (WQDs). Schematic illustration of the synthesis procedure and fabrication process of white luminescent core-shell Cu-Ga-S/ZnS quantum dots.

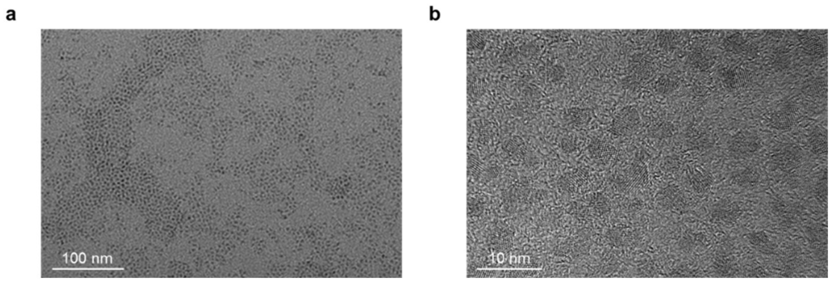

Supplementary Fig. 2│Morphological characterization of WQDs. TEM (**a**) and HRTEM (**b**) images of Cu-Ga-S/ZnS QDs.

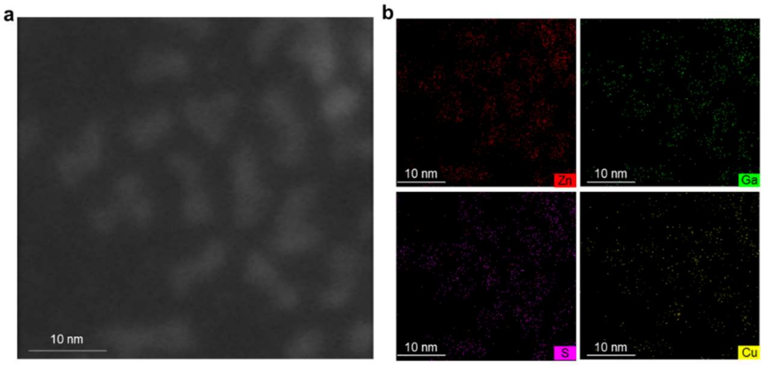

Supplementary Fig. 3 | Element analysis of the WQDs. HAADF-STEM image (**a**) and element mapping (**b**) of Cu-Ga-S/ZnS QDs.

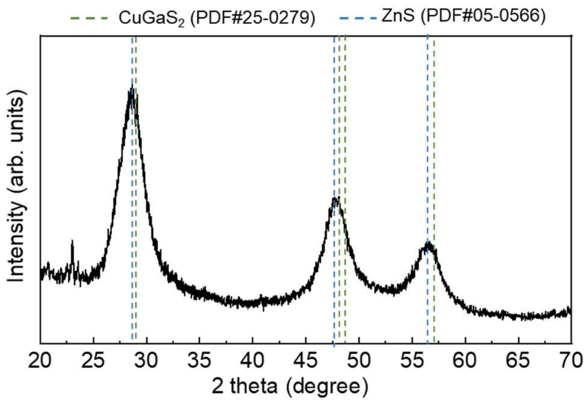

Supplementary Fig. 4 | Crystal structure analysis of the WQDs. XRD pattern of Cu-Ga-S/ZnS QDs.

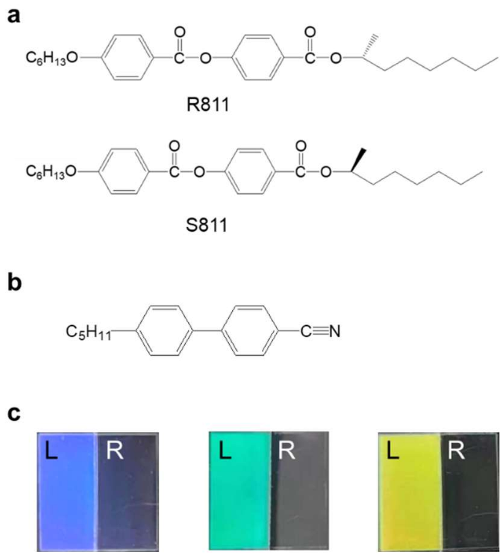

Supplementary Fig. 5 | Structures and structural colors of chiral liquid crystals. a, Molecular structures of chiral dopants R811 and S811. **b**, Molecular structure of the nematic liquid crystal 5CB. **c**, Photographs of the liquid crystal cells under left- and right-handed circular polarizers, respectively, with the weight ratios of R811 in the chiral nematic liquid crystal: 29 wt%, 26 wt% and 23 wt%. Areas of color units (liquid crystal cells) are 4 cm^2. The weight ratios of the chiral dopant could regulate the pitch of the chiral nematic liquid crystal, leading to different structural colors. Right-handed chiral nematic liquid crystal absorbed right-handed light while reflecting the left-handed one, resulting in the obviousness and disappearance of the structural color under left- and right-handed circular polarizers, respectively.

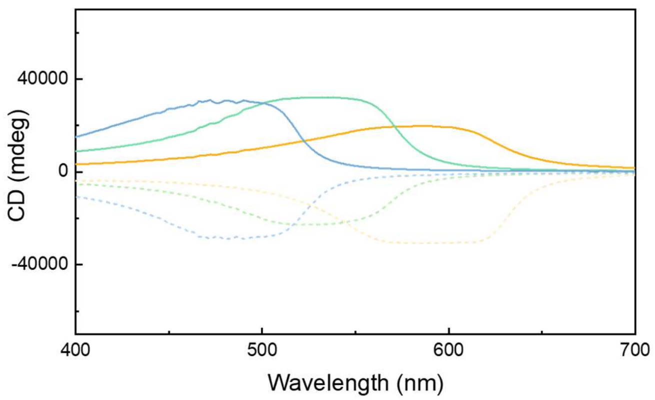

Supplementary Fig. 6│Chiroptical activity characterization. Mirror-imaged CD spectra of spiral full-color emission generator (SFEG) with different weight ratio of S811 (solid curve) and R811 (dashed curve) in 5CB.

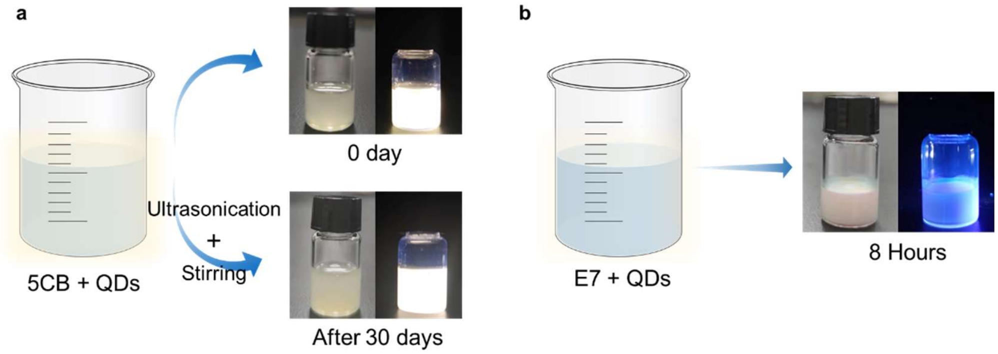

Supplementary Fig. 7 | Compatibility between the chiral liquid crystal and WQDs. a, Images of the WQDs doped chiral liquid crystal 5CB under indoor light (left) and UV light (right) at day 0 and after 30 days with ultrasonication and stirring. **b**, Images of the WQDs doped chiral liquid crystal E7 after 8 h. We took turns using ultrasonication and stirring during the solvent evaporation process, resulting in macroscopically favorable dispersion for more than 30 days in 5CB, while E7 would more severely damage the surface structure of the WQDs, leading to fluorescence quenching and more prone to phase separation.

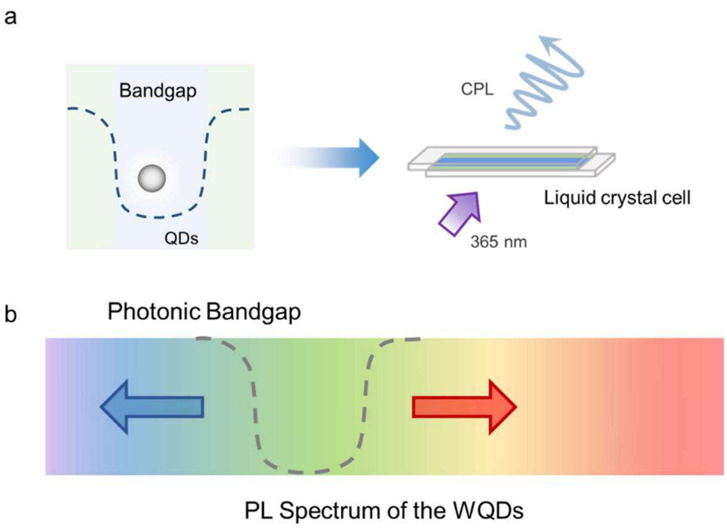

Supplementary Fig. 8 | The photonic bandgap property of the chiral liquid crystal. a, Illustration of the mechanism of photonic bandgap selectivity and generation of CPL. **b**, Illustration of the photonic bandgap of chiral nematic liquid crystal and the PL spectrum of WQDs. We could get stronger CPL signals and larger g_{lum} values when the emitter's PL was embedded within the photonic bandgap of the chiral liquid crystal[6]. Owing to the broad range of the WQDs, the photonic bandgap of the chiral nematic liquid crystal could be coincident with anywhere in the PL spectrum of the QDs without change of order of magnitude in the g_{lum} values.

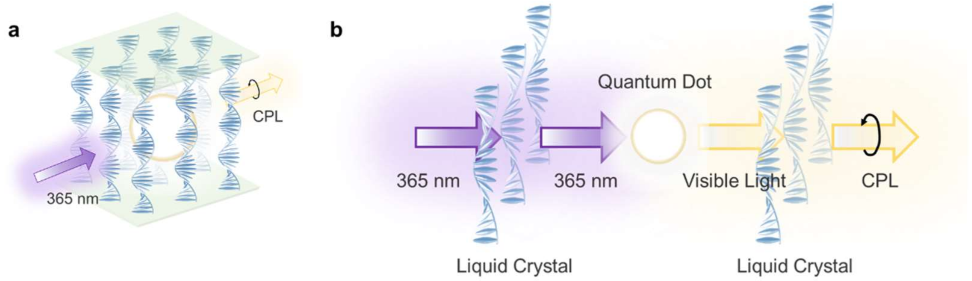

Supplementary Fig. 9 | CPL emission. a-b, Schematic illustration of the CPL emission (**a**) and mechanism (**b**) of SFEG. The UV light first illuminates on the QDs after penetrating the chiral liquid crystal (non-selective absorption of UV light). Then the QDs are excited and the formed photoluminescence passes through the chiral liquid crystal host, during which CPL generates since the chiral liquid crystals could reflect the same handedness CPL and pass the opposite one[6,7].

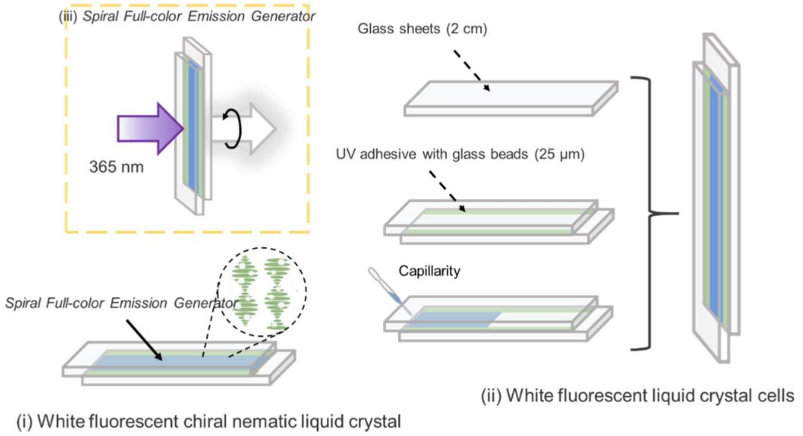

Supplementary Fig. 10 | Fabrication of SFEG-fulfilled liquid crystal cell. The inner cavity thickness of the liquid crystal cell is 25 μm, and then the SFEG could seep into the liquid crystal cell through the capillary phenomenon.

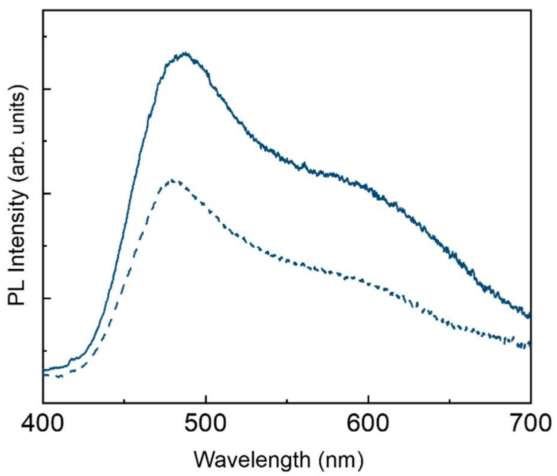

Supplementary Fig. 11 | PL intensity under circular polarizers. PL spectra of left-handed SFEG measured through right- (solid curve) and left-handed (dashed curve) circular polarizers, respectively.

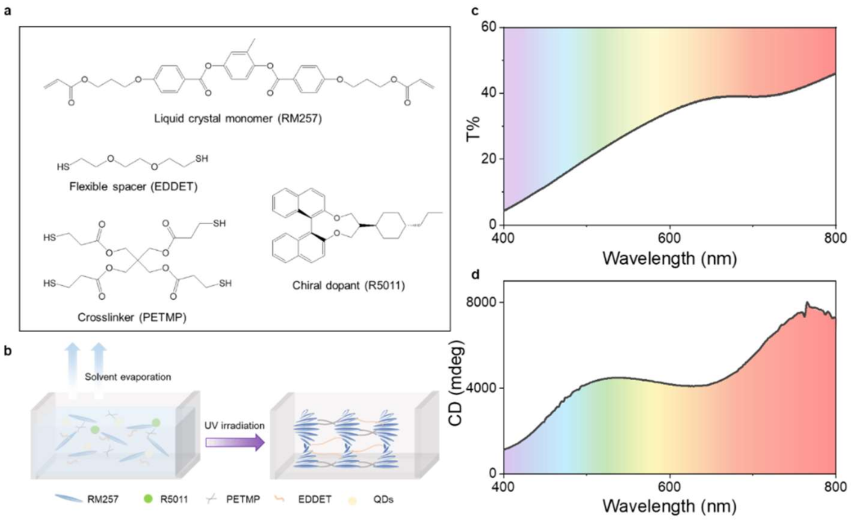

Supplementary Fig. 12 | Synthesis and characterizations of the liquid crystal polymers. a, Molecular structures of the main components of the liquid crystal polymer. **b**, Schematic process of the fabrication of the liquid crystal polymer. **c-d**, Transmission spectrum (**c**) and CD spectrum (**d**) of the liquid crystal polymer. The obtained broad transmission and CD spectra brought inevitability for white CPL.

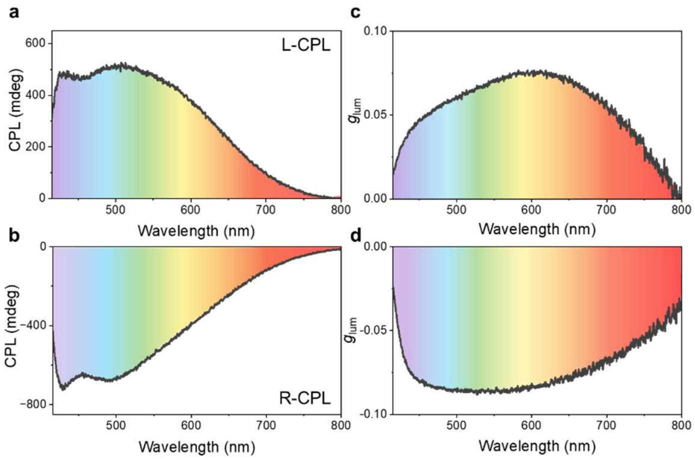

Supplementary Fig. 13 | CPL characterizations of the liquid crystal polymers. a-d, Broad CPL spectra (**a-b**) and the corresponding g_{lum} spectra (**c-d**), generated by combining the liquid crystal polymer with the WQDs.

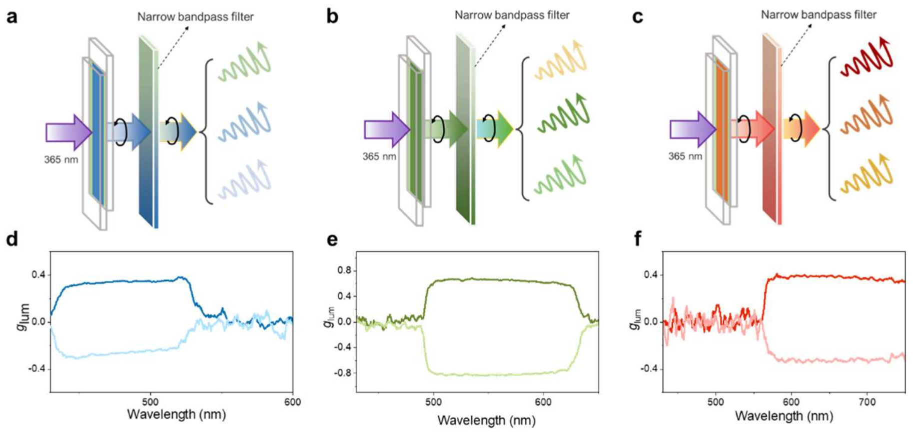

Supplementary Fig. 14│Characterizations of the visualized customized-color CPL. a-c, Schematic illustration of generation of the visualized customized-color CPL. **d-f**, Corresponding g_{lum} spectra of customized-color CPL systems at 455 nm (**d**), 540 nm (**e**) and 633 nm (**f**).

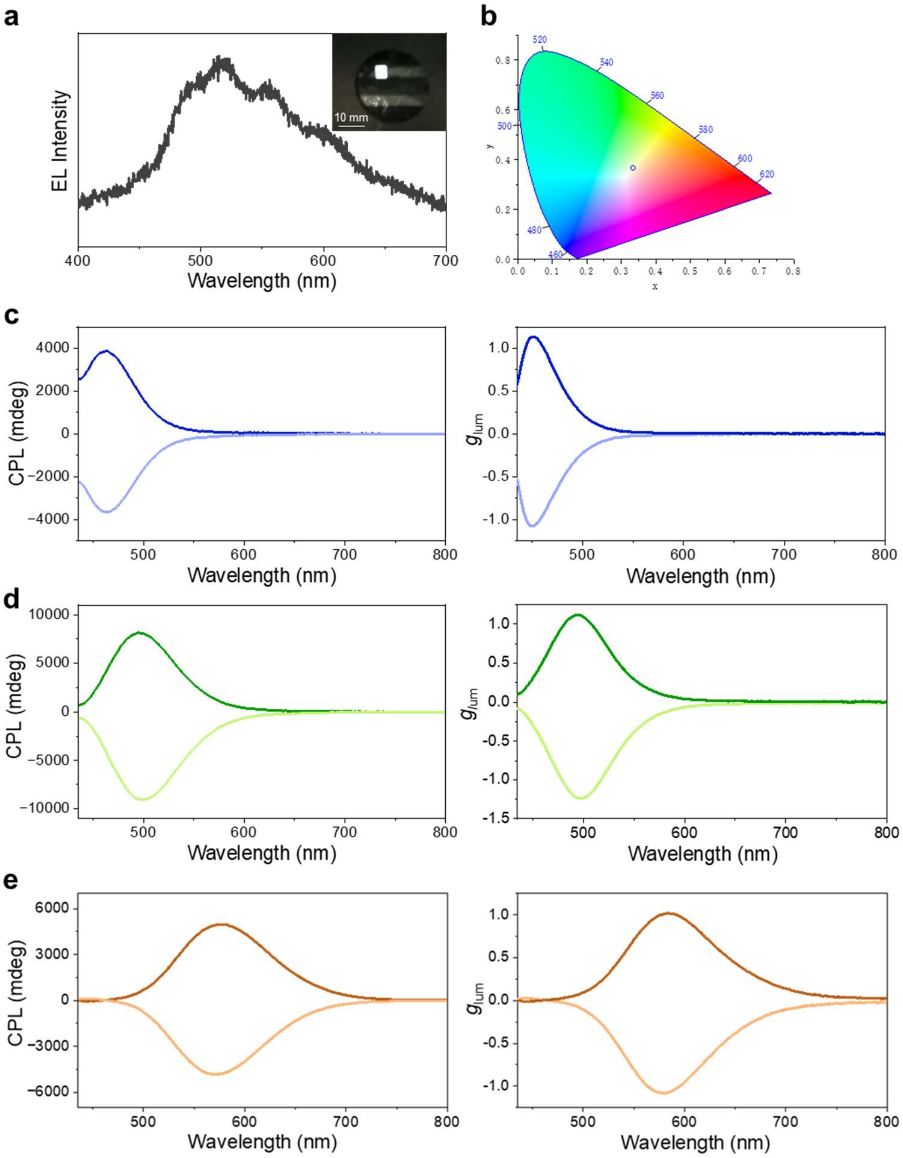

Supplementary Fig. 15 | Characterizations of the white QLED and freestanding liquid crystal films. a-b, Collected EL spectrum (**a**) and the corresponding variation of CIE color coordinates (**b**) of the white QLED. Inset is the bright EL image of the white QLED. **c-e**, Mirror-imaged CPL and the corresponding g_{lum} spectra of the WQDs luminescent layer and the freestanding liquid crystal films with different photonic bandgap in blue, green and red regions, respectively.

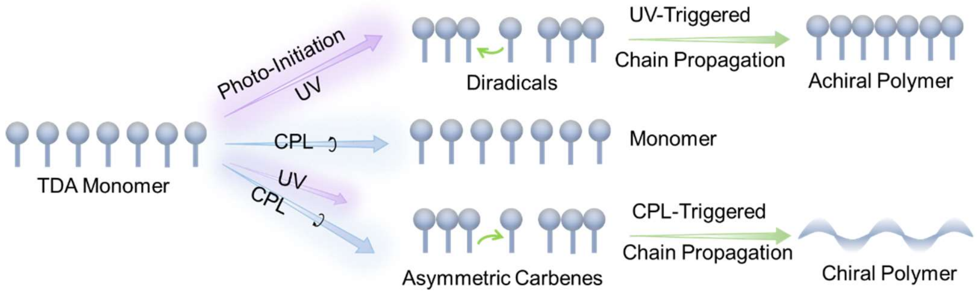

Supplementary Fig. 16│PTDA formation. The photo-initiation of polymerization reaction depends on the excitation of the TDA monomers in the shortwave UV region (< 310 nm), where the TDA monomers could form an oligomer. After that, two ways are considered for the chain propagation. One is that the energy transfers from an excited monomer to the polymer chain triggered by shortwave UV. The other is to excite the oligomer chain directly by CPL when the duplicate units are more than 5. The as-formed asymmetric carbenes might be aligned in a suitable orientation and reacted with the neighboring TDA monomers, eventually leading to the formation of the chiral PTDA chains directed by the handedness of CPL[5].

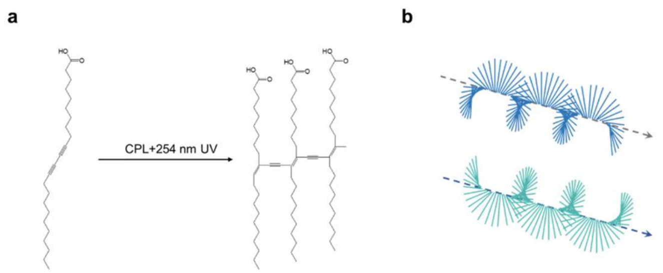

Supplementary Fig. 17│Synthesis of chiral PTDA chains. Illustration of the polymerization process (**a**) and the chiral polymer chains of PTDA (**b**).

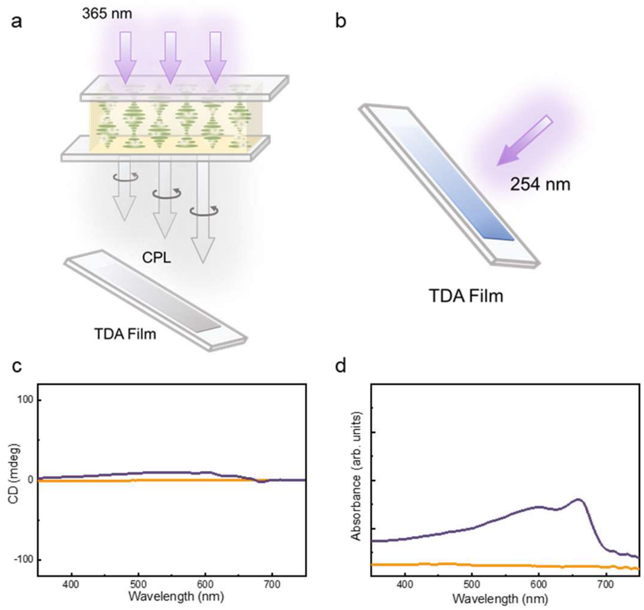

Supplementary Fig. 18 | Schematic illustration showing the enantioselective polymerization of TDA. a-d, Experimental setup for enantioselective photopolymerization of TDA under CPL (**a**) and shortwave UV (**b**). CD (**c**) and UV-vis (**d**) spectra of the chiral PTDA. TDA could not polymerize only under the illumination of CPL, while the PTDA was achiral only under the illumination of shortwave UV light.

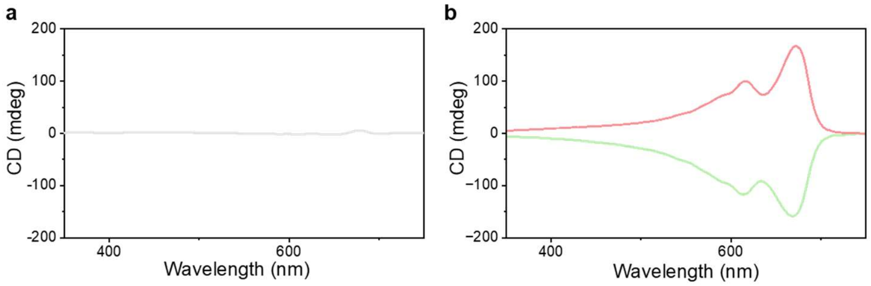

Supplementary Fig. 19 | Characterizations of the polymerization of TDA. a, CD spectra of the PTDA film co-induced by unpolarized light (generated by the WQDs) and shortwave UV. **b**, CD spectra of the PTDA films co-induced by real CPL from commercially-available instrument and shortwave UV. Unpolarized light could not induce the generation of chirality in PTDA, and the CPL generated by circular polarizers could also induce the chirality of PTDA as the CPL from SFEG.

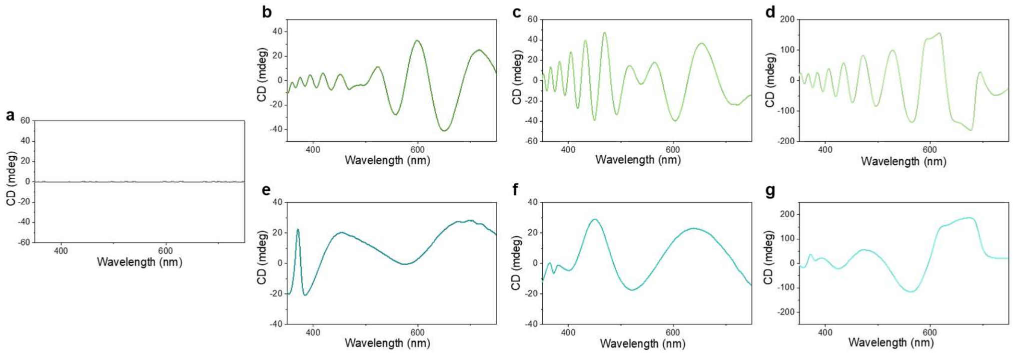

Supplementary Fig. 20 | Characterizations of the enantioselective polymerization of TDA. a, CD spectra of the glass substrate. **b-c**, CD spectra of the PET substrate. **d**, CD spectrum of the PTDA film on the PET substrate. **e-f**, CD spectra of the PS substrate. **g**, CD spectrum of the PTDA film on the PS substrate. The glass substrate showed no CD signal, indicating that the CD signal of the PTDA originated from itself, while the PET and PS themselves showed noisy and uneven CD signals, leading to noisy and unrepeatable CD signals of PTDA.

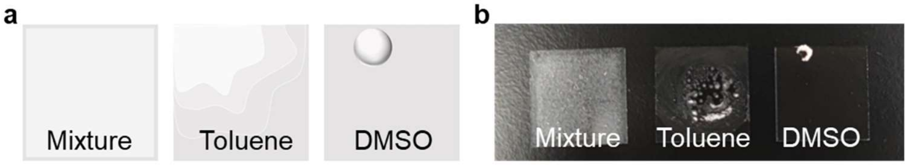

Supplementary Fig. 21│TDA films on the glass with different solvents. a-b, Schematic illustration (**a**) and images (**b**) of the TDA films with different solvents (0.2 mL) on gasses: mixture of ethanol and deionized water (ethanol: deionized water = 4:1, left), toluene (middle) and DMSO (right). The mixture of ethanol and deionized water led to uniform films on glasses, while using toluene and DMSO as solvents could not produce uniform films.

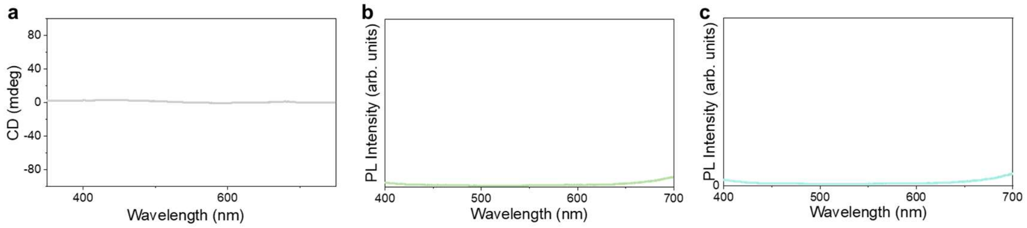

Supplementary Fig. 22 | Optical characterizations of the TDA and PTDA. a, CD spectrum of the TDA monomer film. **b-c**, PL spectra of the TDA monomer (**b**) and the chiral PTDA (**c**). TDA itself is achiral and TDA and PTDA are both lightless.

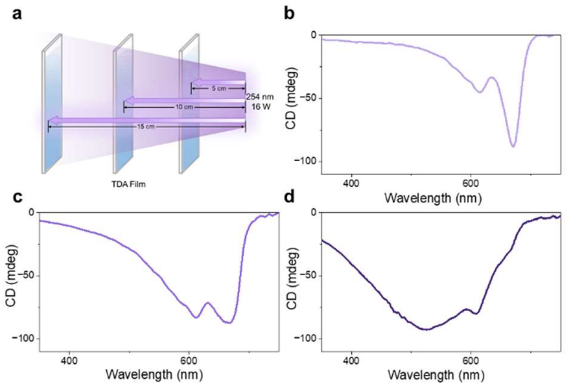

Supplementary Fig. 23 | Characterizations of the enantioselective polymerization of TDA with different light intensity. a, Schematic illustration of the light intensity generated by the UV lamp (254 nm, 16 W), regulated via the distance between the sample and light source. **b-d**, Corresponding CD spectra of the PTDA films with tuning sample-light distances, 15 cm (**b**), 10 cm (**c**) and 5 cm (**d**). With decreased distance, the polymerization became faster and more difficult to control and the corresponding CD spectra showed blueshifts and broadening. We chose the distance of 15 cm to carry out the enantioselectivity experiments.

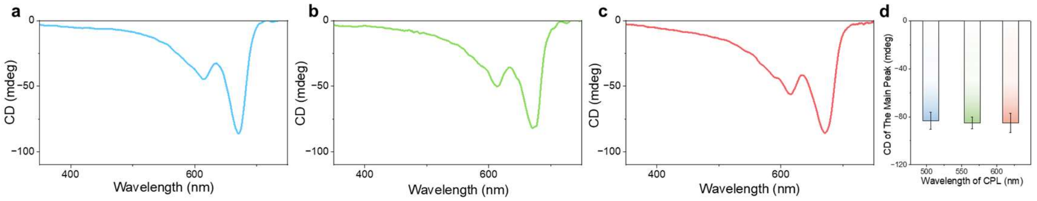

Supplementary Fig. 24 | Characterizations of the enantioselective polymerization of TDA with different batches. a-c, CD spectra of the chiral PTDA induced by right-handed blue (500 nm) (**a**), green (560 nm) (**b**) and red (620 nm) (**c**) CPL generated by SFEG with regulated photonic bandgap with the assistance of shortwave UV (the sample-light distance is 15 cm). **d**, Different batches (n = 3) of the PTDA films co-induced by right-handed CPL generated from SFEG and shortwave UV. The wavelength of the CPL in the visible light region had no significant effect on the chirality of the PTDA films, and the CD spectra of different batches of the samples indicated fine repeatability of the photoinduced polymerization.

Supplementary Table 1 | Results of the enantioselective polymerization of TDA.

Content	Variable	Result
Polarization of light	Unpolarized light	No CD signal
	Circularly polarized light	Opposite CD signal
	CPL from polarizers	Opposite CD signal
Substrate	Glass	Opposite CD signal
	PET	Noisy CD signal
	PS	Noisy CD signal
Solvent	Mixture of DI water and ethanol	Well-distributed film
	Toluene	Uneven surface
	DMSO	Uneven surface
CD spectrum	TDA monomer	No CD signal
	PTDA	Opposite CD signal
CPL spectrum	TDA monomer	No CPL signal
	PTDA	No CPL signal
UV light power	15 cm	CD signals
	10 cm	Slight blue shift CD signal
	5 cm	Widened CD signal
Batch effect	1	Fine repeatability
	2	Fine repeatability
	3	Fine repeatability

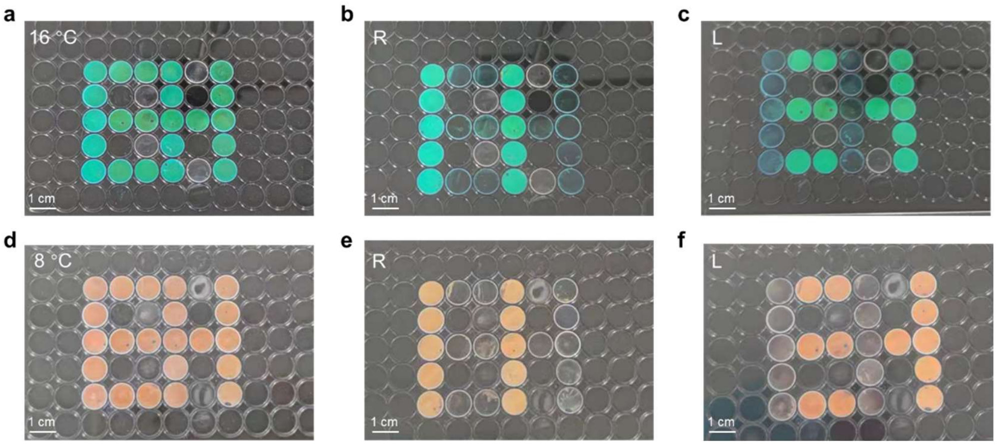

Supplementary Fig. 25 | Information encryption and anti-counterfeiting based on the SFEG. **a-c**, The pattern formed by the SFEG (with 26 wt% chiral dopants S/R811) showed green color at ca. 16 °C and provided information (color unit) change under natural light (**a**), right- (**b**) and left-handed circular polarizers (**c**), respectively. **d-f**, The pattern color changed to red when the temperature decreased, as well as giving distinctive information observed by different polarizers.

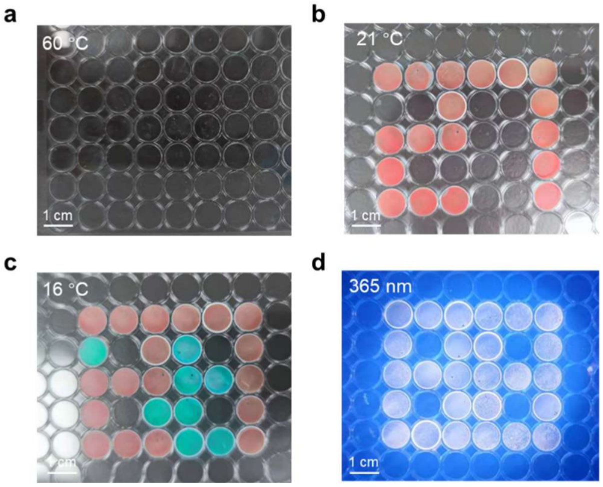

Supplementary Fig. 26 | Information responses with different stimuli in the SFEG system (with different weight ratios of the chiral dopants). a, The color-unit-composed pattern became transparent at a high environmental temperature (here, it was 60 °C). **b-c**, The pattern changed from red (**b**) to mixed color (**c**) mediated by temperature decrease. **d**, Bright-white color emission achieved by 365 nm excitation.

Supplementary References

1. Ma, J. *et al.* Mechanochromic, sape-programmable and self-healable cholesteric liquid crystal elastomers enabled by dynamic covalent boronic ester bonds. *Angew. Chem. Int. Ed.* **61**, e202116219 (2022).
2. Guo, Q. *et al.* Multimodal-responsive circularly polarized luminescence security materials. *J. Am. Chem. Soc.* **145**, 4246–4253 (2023).
3. Kim, J. H. *et al.* White electroluminescent lighting device based on a single quantum dot emitter. *Adv. Mater.* **28**, 5093–5098 (2016).
4. Han, H. *et al.* High-performance circularly polarized light-sensing near-infrared organic phototransistors for optoelectronic cryptographic primitives. *Adv. Funct. Mater.* **30**, 2006236 (2020).
5. Wang, X. *et al.* Circularly polarized light source from self-assembled hybrid nanoarchitecture. *Adv. Opt. Mater.* **10**, 2200761 (2022).
6. Yang, X., Zhou, M., Wang, Y. & Duan, P. Electric-field-regulated energy transfer in chiral liquid crystals for enhancing upconverted circularly polarized luminescence through steering the photonic bandgap. *Adv. Mater.* **32**, 2000820 (2020).
7. Liu, S. *et al.* Circularly polarized perovskite luminescence with dissymmetry factor up to 1.9 by soft helix bilayer device. *Matter* **5**, 2319–2333 (2022).
